# X-ray computed tomography to study rice (*Oryza sativa* L.) panicle development

**DOI:** 10.1093/jxb/erv387

**Published:** 2015-08-11

**Authors:** Vibhuti M. Jhala, Vrinda S. Thaker

**Affiliations:** Plant Biotechnology and Molecular Biology Laboratory, Department of Biosciences, Saurashtra University, Rajkot, 360 005, Gujarat, India

**Keywords:** CT scanning, Hounsfield unit (HU), rice (*Oryza sativa* L.).

## Abstract

Computational tomography is an important technique for developing digital agricultural models that may help farmers and breeders for increasing crop quality and yield. In the present study an attempt has been made to understand rice seed development within the panicle at different developmental stages using this technique. During the first phase of cell division the Hounsfield Unit (HU) value remained low, increased in the dry matter accumulation phase, and finally reached a maximum at the maturation stage. HU value and seed dry weight showed a linear relationship in the varieties studied. This relationship was confirmed subsequently using seven other varieties. This is therefore an easy, simple, and non-invasive technique which may help breeders to select the best varieties. In addition, it may also help farmers to optimize post-anthesis agronomic practices as well as deciding the crop harvest time for higher grain yield.

## Highlight

The computed tomography (CT) scanning method is used for the temporal analysis of panicle development in two rice varieties varying in their yield, in order to understand seed development and its correlation with growth parameters.

## Introduction

Rice (*Oryza sativa* L.) is an important food crop for over half the human population. Breeding for better quality and high-yield is the main focus of rice geneticists and breeders. There is a great demand for improving grain quality and quantity to meet the requirements of an increasing world population. Understanding the fundamental genetic factors that control rice yield, together with supporting the breeders’ ability to manipulate such factors, would be an added advantage for ensuring an adequate global food supply. The number of seeds per panicle is believed to be the most plastic yield component, whereas the panicle number and individual seed weight are under more rigid genetic control (Zhikang *et al.*, 1997; Luo *et al.*, 2001). Yield in rice can be calculated as the number of seeds per panicle, the size of seeds, the number of panicles per plant, and the number of plants per hectare. The arrangement of seeds in a panicle is central to the growth of the seeds and the yield of the crop. Many agronomical and/or physiological studies have been conducted to judge the varietal potential of different plants and the correlation of growth parameters in yield determination (Koutroubas and Ntanos, 2003; Prasad *et al.*, 2006; Li *et al.*, 2012). These studies give a reasonable understanding of seed development and yield determination; however, they are time-consuming and require tedious methodologies. In addition, the separation of seeds from the panicle, followed by an estimation of seed size and position within panicle restrict the sample size for an evaluation of crop yield.

High through-put, non-destructive phenotyping methods provide an opportunity to analyse the growth and development of plants with accuracy and speed (Yang *et al.*, 2013, Humplík *et al.*, 2015). High-resolution X-ray computed tomography (CT) scanning has the possibility of visualizing the panicle in 3-D space *in situ* because it is a non-invasive and non-destructive procedure (Kalender, 2000). In addition, continuous sectioning of an object can be skeletonized into 2-D images for detailed analysis. In many plant experiments, the CT scanned data are used to study root architecture in soil that may help researchers to understand root growth and development in relation to its geological conditions (Grose *et al.*, 1996; Moradi *et al.*, 2011; Schluter *et al.*, 2010).

Rice functional genomic research using various genomic tools is a prime focus because of its importance for understanding a model monocot genome. In addition, in order to fill the gaps between genomic analysis and breeding for better quality high-yielding rice, automated phenotyping methods are required to replace the currently practised manual ones (Duan *et al.*, 2011). In recent years, the number of tillers per plant have been calculated using CT scanning in rice (Yang *et al.*, 2011). However, to the best of our knowledge, no such study for panicle development has been reported. Rice panicle development over time was studied here using CT scan technology to understand yield potential in rice together with exploring its use for the best possible agriculture practices. Two rice varieties were selected that differed in their final yield in order to understand how the CT scan parameters are related to the yield difference. These data were upgraded and analysed by other optional analytical procedures and the results for two rice varieties at different developmental stages are reported. The CT scanning configurations were adjusted to optimize data collection for the different stages from initial seed setting to the mature seed. The procedures to construct a 3-D image of the panicle from the CT scan data, which were then skeletonized to 2-D images, are described. Briefly, an attempt was made to observe a correlation between the HU value and growth parameters using X-ray computation tomography of the rice panicle. This correlation was substantiated by similar analyses in seven different rice varieties at maturity. It is hoped that this approach may act as a guide for deciding the appropriate harvest time.

## Materials and methods

### Plant sampling

Two hybrid varieties of rice (*Oryza sativa*), PAC801 and PAC807, were grown and maintained at the Vikram farm, Vapi, Gujarat (20.3667 °N, 72.9000 °E). The former is higher yielding compared with the latter. The normal cultural practices such as irrigation, the applications of fertilizers, insecticides, pesticides etc. were conducted to optimize the yield. On the day of anthesis, uniformly growing panicles were tagged for each variety and harvested at the desired periods. The number of panicles per plant and plant height were noted for 100 plants and the mean values ± standard deviation (SD) are shown here.

### Growth analysis

For the measurement of fresh and dry weights, the tagged panicles were harvested at weekly intervals. The seeds were separated, counted, and weighed before and after oven-drying at 60 °C until a constant weight was recorded. Seed water content was calculated by differences in fresh and dry weights. Data were collected in triplicate and the mean dry weights and water content are reported. The data were fitted to polynomial equations for a best-fit curve for each data set. Changes in the value per unit time were calculated as rate for a parameter and plotted against days after flowering (DAF). ANOVA (single factor) was performed for data on dry matter accumulation (DMA) and water content.

### Computational tomography (CT) scanning

CT scanning technology, routinely used with X-rays to visualize thin cross-sections of an organ, was used here for rice panicles. X-rays are generated from a source located to one side of the gantry, attenuated through the panicle, and then registered by a series of detectors placed on the opposite side of the panicle. Both X-ray source and detectors make a synchronous movement around the panicle so that all angles of measurement are covered. As the X-ray penetrates through a panicle, some X-rays are absorbed. The higher the density of a part of the panicle, the higher is its X-ray absorption.

In a preliminary experiment, a set of scanning configuration parameters was optimized for standardization (see Supplementary Fig. S1 at *JXB* online). In the first season, the growth stages were determined and standardized for the CT scanning procedure. In the second season, the HU value and growth were determined for the two varieties and their relationship was developed. This relationship was further confirmed with seven other varieties by measuring CT scan parameters at maturity stage.

### Image collection

Panicles from the two rice varieties, collected at different developmental stages, i.e. initial cell division (5 d), milky white (10 d), dry matter accumulation (15 d), and maturation stage (25 d), were subjected to CT scanning.

No special preparation of the samples was required for CT scanning. The collected panicles of both varieties were scanned individually in a Philips high-resolution medical CT scanner (GE Medical Merge), installed at a private Medical Centre: Prabhat CT Scanning Hospital, Rajkot, India. The images collected were visualized in CT scan software eFilmLite. The window/level (W/L) adjustment for rice panicles was the first step for proper visualization of the images. The panicles were best observed in 1500/–600 (lungs Window/level). All the scanned images were pre-processed in the Merge Healthcare CT scanner software utilizing the Multi-Planar Reformatting (MPR) parameter (Instrument software). Sagittal (dividing into left and right pieces) and coronal (dividing into anterior and posterior pieces) Multi-Planar Reformatting (MPR) provides easier visualization by projecting two orthogonal viewing planes from a 2-D image. The MPR tool gives an arbitrary perpendicular viewing plane from a 2-D image. By using the MPR, different views were created and used for determining Hounsfield unit (HU), for panicle length and seed size measurement.

### Hounsfield unit (HU) and seed size determination

The cross-sectional images constructed from the CT scan data are maps of CT numbers (CTN), which are an average of the relative measurement of the pixel density in the image, also known as the corresponding volume or ‘voxel’. The CTN values are calculated using the X-ray linear attenuation coefficient, and the unit of CTN is the Hounsfield unit (HU), briefly calculated as,

$$\text{CTN }\left(\text{HU}\right)=\mu_{\text{object}–}\mu_{\text{water}}/\mu_{\text{object}–}\mu_{\text{air}}\times \text{1}000$$

Where, μ_object_ is the mean value of linear attenuation coefficient for the voxel; μ_water_ is the linear attenuation coefficient of pure water; and μ_air_ is that of a standardized air sample (Kalender, 2000). The CTN scale is linear and has a central axis at 0, which corresponds to water, while air has the calibrated value of –1 000 HU. Hence, for the CTN values, the intensities received by the detectors are expressed in the linear Hounsfield unit (HU) scale for which typical values are –1 000 for air and 0 for water.

The CTN values were derived for each processed image scan. Each panicle was divided into three equal zones; upper, middle, and lower. A total of 100 seeds from each scan were used for HU value and the calculation of seed size. The measurement of seed size was done using the in-built MPR tools (see Supplementary Figs S2 and S3 at *JXB* online).

### Pseudo-colouring of images

ImageJ ver.1.46 software (http://imagej.nih.gov/ij) was used for the pseudo-colouring of all the images. The pseudo-colouring option of the software (the application of 16 colours) was applied for a better visualization of the panicles. A pseudo-colour (or indexed colour) image is a single channel grey image (8-, 16-, or 32-bit) that has colour assigned to it via a look-up table in ImageJ. The grey values of ImageJ match red, green, and blue values in such a way that the shades of grey are displayed as coloured pixels: 8-bit grey-coloured images were turned into a 16-colour image in order of their developmental stages.

## Results and discussion

### Growth analysis

**Fig. 1.**
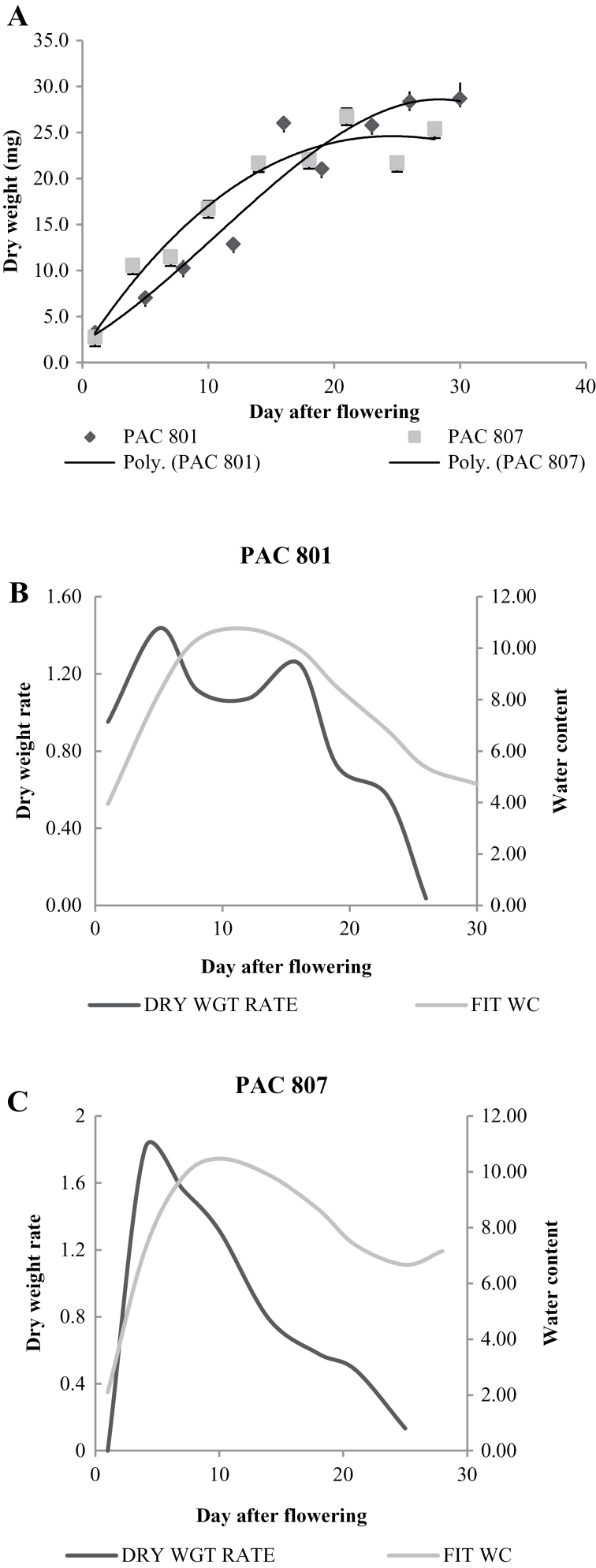
(A) Change in mg dry weight. (B) Water content (WC) and rate of dry matter accumulation (DMA) in variety PAC801 and (C) PAC807 against seed age in days. Each data point represents the mean value of 25 replicates at a given age; ± represents the standard deviation of the sample or otherwise within the symbols.

The plant heights of PAC801 and PAC807 were almost equal at 125–130 cm: however, the duration of vegetative growth was longer (20 d) in PAC801 than in PAC807. In PAC801, the number of seeds per panicle was also greater (170–215) than in PAC807 (165–200). In addition, the number of tillers was greater in PAC801 (30–32) than in PAC807 (25–30) (Table 1). At a given time, if the number of plants per unit area was equal, the final yield was higher in PAC801. Data of seed dry weight and water content were fitted to polynomial equations of different degrees, and the best-fit equation was determined statistically by performing a *t* test for different *r* ^2^ values. The data on DMA were adequately explained by a 3rd degree polynomial equation (Fig. 1A). Seed dry weight, started accumulating after fertilization, and entered the linear phase of DMA up to 30 DAF. Maximum DMA was recorded in PAC801 (27mg) and PAC807 (21mg) on 28 DPF. In subsequent periods, no significant change in DMA was observed in both varieties.

**Table 1.** Yield component data of two rice varieties

Characters	PAC-801	PAC-807
Grain yield (tn/ht)	10.1764	9.9788
Height (cm) *n*=10	124±4	121.5±3.5
No. of seeds/spike *n*=10	192.5±45	182.5±35
No. of tillers/plant *n*=10	31±1	27.5±2.5
Seed weight (mg) *n*=200	33.13	31.9

The rate of DMA per day reached a maximum on day 5 in both varieties and declined thereafter. The declining trend was very sharp in PAC807, whereas in PAC801 the value remained higher up to 18 d and declined thereafter (Fig. 1B, C). The rate of dry matter accumulation is one of the parameters that determine the final yield (Bewley and Black, 1985). Data on water content revealed a gradual increase in both the varieties, but initially a higher value was observed in PAC801. The maximum value was almost similar (12mg) in both. However, like the rate of DMA, the duration was longer in PAC801 than PAC807 (Fig. 1B, C). Water status has been reported to play an important role in the development of cotton (Rabadia *et al.*, 1999), wheat seeds (Chanda and Singh, 1998), soybean and other crops (Egli, 1990; Egli and Tekrony, 1997). In cereal crops, grain-filling is a critical and dynamic process that determines the final grain yield (Borra and Westgate, 2006). Seed filling is sensitive to water shortages as the water relations of the developing seed plays a fundamental role in seed filling (Schnyder and Baum, 1992).

In this experiment, single factor ANOVA for DMA and WC data of both the varieties were statistically significant; although that of PAC801 were more significant (*P* ≤0.001) than PAC807 (*P* ≤0. 01). From these data analyses, rice seed development was divided into (i) a cell division phase (0–5 d), (ii) a cell elongation/milky stage (5–12 d), (iii) DMA (10–25 d), and (iv) a maturation phase (25 d onward).

**Fig. 2.**
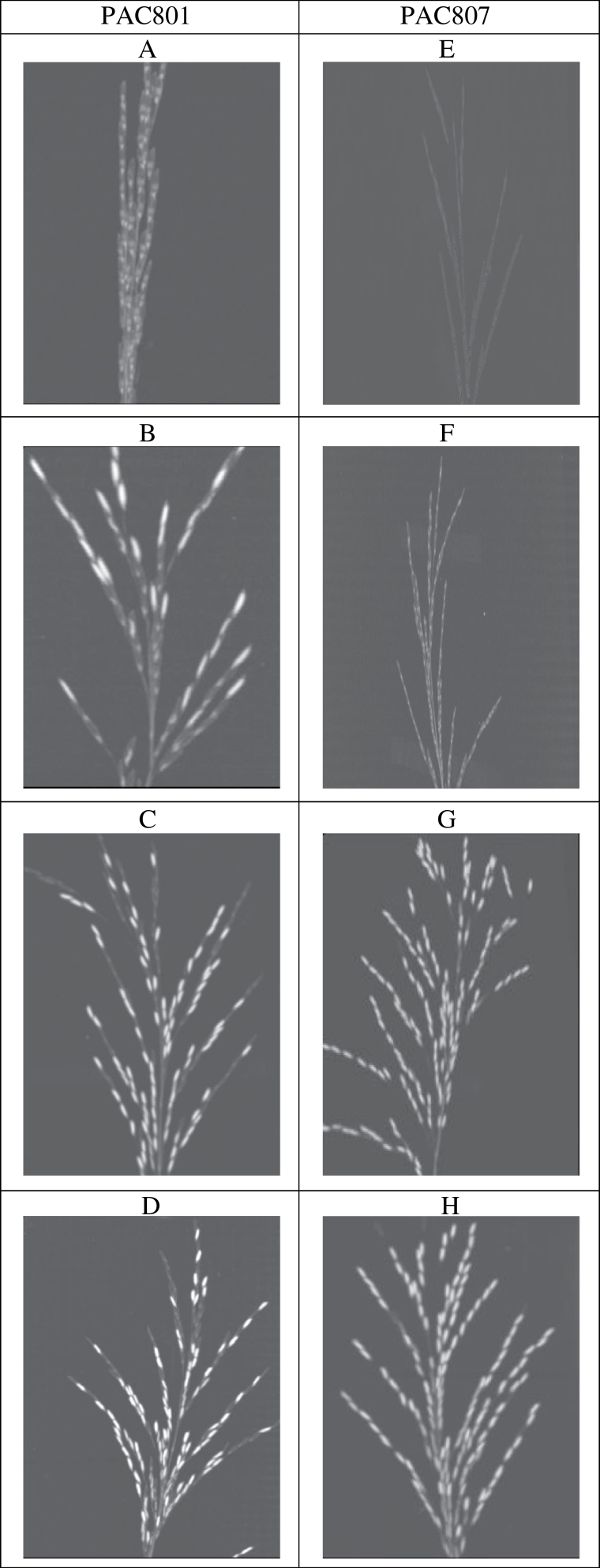
Spikelet images of rice varieties PAC801 (A–D) and PAC807 (E–H), at the initiation (A, E), elongation (B, F), dry matter accumulation (C, G), and maturation (D, H) phases, respectively. CT scan image is in the Multi-Planar Reformatting (MRP) tool to visualize images for calculating various topological parameters (for other details see Supplementary Fig. S1 at *JXB* online).

Panicles of rice were scanned separately for PAC801 and PAC807 at the four stages. The panicle was placed on the bed of the CT scanner and moved vertically through the X-ray plane under the conditions described earlier (Table 2). A total of 177–344 images for PAC801 and 144–287 images for PAC807 were generated covering 10cm (initial) to 25cm (mature) panicle length of the respective varieties. Images were placed in a 2-D view of the complete panicle and 10 images were selected from the centre (Fig. 2; Table 2).

**Table 2.** Image collection from two rice varieties at stages of developing seeds CT scan conditions: voltage at 120kV, current 50 mA, scanning thickness 1.5mm, voxel 0.2621mm^3,^ Mode Tomo and child head programme.

Stages	Initiation	Elongation	Dry matter accumulation	Maturation
Scan spike length (cm)	10	12	20	22
Image numbers: PAC-801	144	216	201	287
PAC-807	173	173	287	344

### Calculation of HU value

**Fig. 3.**
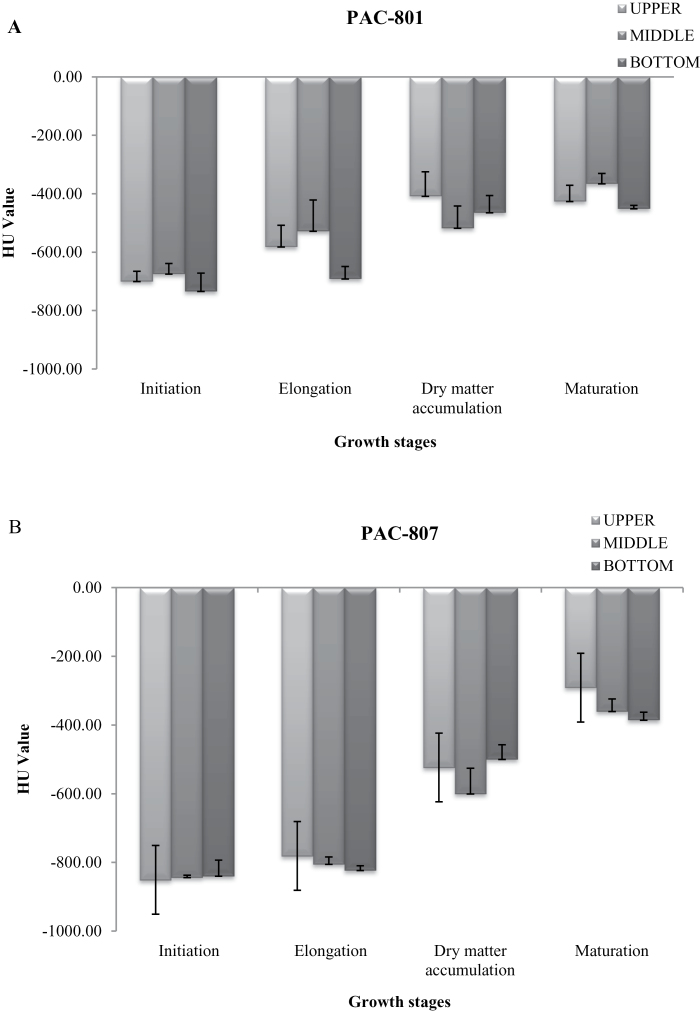
Changes in HU value in PAC801 (A) and PAC807 (B) at different growth stages of the seed: (1) initiation, (2) elongation, (3) dry matter accumulation, and (4) maturation. The upper, middle, and bottom bars indicate the position of the seeds on the panicle from the top to the bottom, respectively. The vertical bars represent ±std of the mean of 100 replicates.

The CT scanned images were opened in the MRP software for further analysis. In the first screening, a total of 10 images was selected from the centre for detailed analysis. In rice panicles, grain development is asynchronous (Mohapatra *et al.*, 1993; Ishimaru *et al.*, 2003). The degree and rate of grain filling among spikelets can be very different according to their position on a panicle. Thus from the images, the seeds were selected from the upper, middle, and lower parts (see Supplementary Fig. S2 at *JXB* online). The CTN scale was linear and centred on 0 (zero) which corresponds to water. Positive and negative values represent matter denser and less dense than water, respectively. Air has a calibrated CTN value of –1 000 HU. One image is generated from a single 512×512 matrix of CTNs for a given interval along the subject. The image represents the cross-sectional volume of the subject. Accordingly, a CT image is a map of relative density (Kalender, 2000). There is a direct relationship between CTN and material density. From these images of panicles, 100 seeds were used for the HU value and the determination of seed size. Mean HU values and size were calculated in all three parts of both the varieties for the different developmental stages (Fig. 3). More negative HU values were observed during the early stage of seed development and moved towards more positive when seeds reached maturity. This pattern was similar in both the varieties. In addition, little difference was observed in HU value with position on the panicle. The value ranged from –200 to –750 in the rice varieties studied. At the time of seed set, water content decreased with seed maturation; and when the seed DMA stabilized, the water content was negligible (Fig. 1B, C). Thus the pattern of HU value and seed water content also showed a relationship. A similar decrease in water content with wood density and HU value has been reported by Wu *et al.* (2011). The data analysed for HU value and seed dry weight using single factor ANOVA also showed statistically significant results (*P* ≤0.001) for both varieties. The principal advantage of a direct relationship between HU value and water content is that it can be used to understand the seed water status during the developmental stages which is a guiding point for the time of harvest and for fertilizer application, which is a tedious process to estimate manually. It has been demonstrated in a number of studies that water content and seed dry matter accumulation rate are directly correlated (Rabadia *et al.*, 1999; Egli and Tekrony, 1997). Since the amount of water is also related to the rate of dry matter accumulation in the rice varieties in the present study and elsewhere, it should also provide information for screening better yielding varieties for the breeders.

**Fig. 4.**
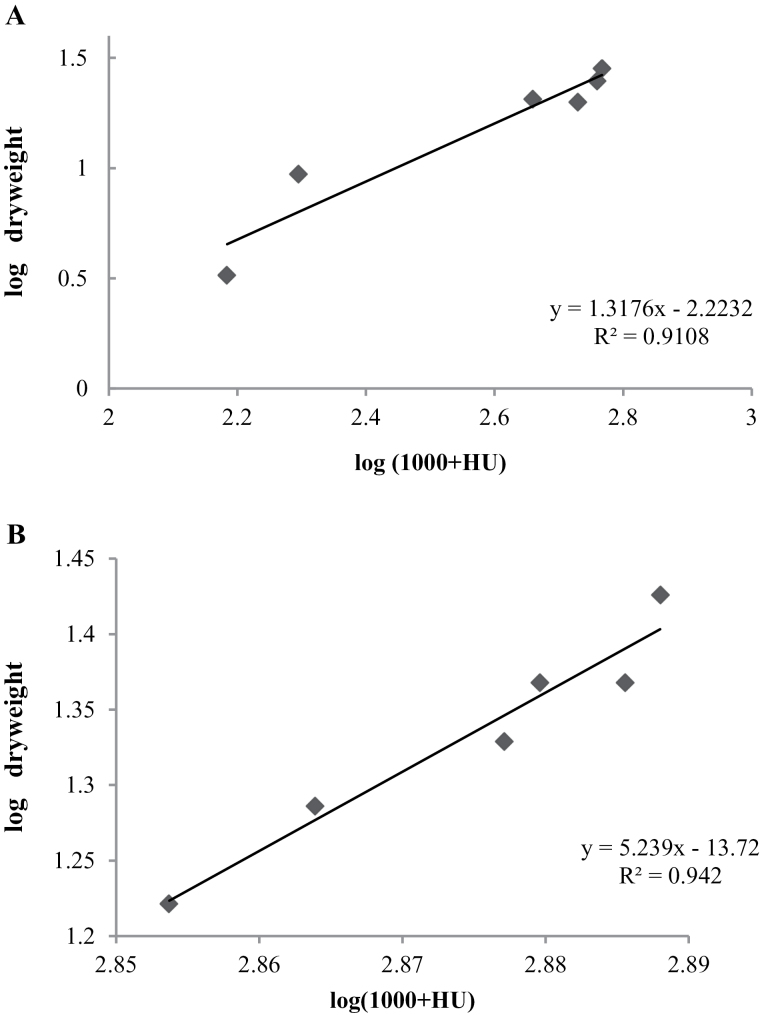
(A) Pooled linear relationship between mean log dry matter accumulation versus log (1 000+HU value) at different growth stages of seed development in PAC801 and PAC807, rice varieties (*n*=200). (B) Linear relationship between mean log dry matter accumulation versus log (1 000+HU value) in seeds of seven different rice varieties (*n*=175); other details as in Table 1.

In particular, HU values and seed dry weight data can be better explained by a linear relationship of log (1 000+HU value) versus log dry weight data. The calculation for dry weight at different growth phases and their respective HU values showed a linear relationship (Fig.4A; *r* ^2^=0.910, *n*=800). The above experiment was repeated with seven other rice varieties which also showed the linear relationship (Fig. 4B; *r* ^2^=0.942, *n*=175), thus the authors believe that it may help breeders to screen for promising rice varieties.

**Fig. 5.**
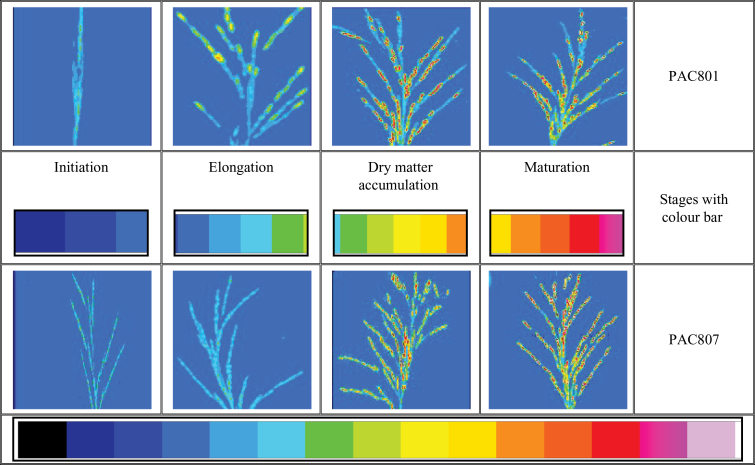
Pseudo-colouring effect on the 8-bit binary CT scan images of PAC801 and PAC807 with developmental stages by using ImageJ software. Changes from the blue to the red scales indicate changes in water content and dry matter at the different stages of seed development. (The stronger the blue colour, the higher the seed water content; the stronger the red colour, the higher the dry matter accumulation in the seed.)

Pseudo-colouring simulates the architecture of seed development studies as well as their image analysis with the developmental stages. It is easy to trace more or less densely packed seeds in the panicle by viewing the contrasting images (Fig. 5). This pseudo colouring approach could further help in evaluating good varieties. The changes in colour pattern, i.e. initially blue to yellow-red, indicates that water content decreases with seed age and this may help to predict harvest time and seed density.

## Conclusion

In the present study, CT scanning has been used to analyse rice panicle development in two rice varieties that differ in their yield potential. The HU value showed a close correlation between water content and dry matter accumulation in seeds at different developmental stages. The correlation between HU values from CT scans and seed development in the panicle suggests that this technique could be useful for farmers and breeders. For example, CT scanning, together with the pseudo-colour development method, can help to understand plant demand during the post-anthesis period. The HU value may be taken as a marker to improve agricultural practices such as irrigation, the application of growth-promoting substances, and developmental status at harvest time and so may help farmers to decide when to harvest the standing crop. All this is possible in a predictable and non-invasive manner. In addition, it is also hoped that this technique may help variety selection in breeding programmes. The authors are aware of the fact that the usefulness of this technique in variety selection requires more work in terms of larger data sets of different varieties and may, perhaps, require some modification of the technique in order to develop a robust system. This will be the focus of our future work.

## Supplementary data

Supplementary data are available at *JXB* online.

A schematic diagram of CT scan procedure for rice panicle.Figure S1. (A) eFilmLite CT scan software, (B) W/L (window / level) adjustment, (C) MPR (MultiPlanar Reformatting) tool and (D) Series of sections observed and Export selected image.Figure S2. Calculation of HU value from the MPR images, (A) PAC801 and (B) PAC807. Rice panicle axis divided equally in to three part: upper, middle and lower in both the varieties (individual white portion in Figure indicates seeds).Figure S3. Seed size measurement in eFilmLite CT software (A) PAC801and (B) PAC 807.Table S1. Seven rice varieties used in study.

Supplementary Data

## Abbreviations:

CT: computational tomography
DAF: days after flowering
DMA: dry matter accumulation
HU: Hounsfield unit
MPR: Multi-Planar Reformatting
W/L: window/level.
