# Supplementary material for: X-ray computed tomography to study rice (Oryza sativa L.) panicle development
Source: J Exp Bot. 2015 Aug 11;66(21):6819–25. doi: 10.1093/jxb/erv387 (PMC4623690; doi:10.1093/jxb/erv387)
Supplement: Supplementary Data [file supp_66_21_6819__index.html]

X-ray computed tomography to study rice (Oryza sativa L.) panicle development — Supplementary Data 

# X-ray computed tomography to study rice (*Oryza sativa* L.) panicle development

## Supplementary Data

Data files

- Supplementary Data - Supplementary Data
