# Supplementary material for: X-ray computed tomography to study rice (Oryza sativa L.) panicle development
Source: J Exp Bot. 2015 Aug 11;66(21):6819–25. doi: 10.1093/jxb/erv387 (PMC4623690; doi:10.1093/jxb/erv387)
Supplement: Supplementary Data [file supp_erv387_Supplementary_materials_caption.pdf]

Supplementary materials caption:

A schematic diagram of CT scan procedure for rice panicle

Supplementary Fig: 1 (A) eFilmLite CT scan software, (B) W/L (window / level) adjustment, (C) MPR (MultiPlanar Reformatting) tool and (D) Series of sections observed and Export selected image

Supplementary Fig: 2 Calculation of HU value from the MPR images, (A) PAC801 and (B) PAC807. Rice panicle axis divided equally in to three part: upper, middle and lower in both the varieties (individual white portion in Figure indicates seeds)

Supplementary Fig: 3 Seed size measurement in eFilmLite CT software (A) PAC801and (B) PAC 807

Supplementary Table: 1 seven rice varieties used in study

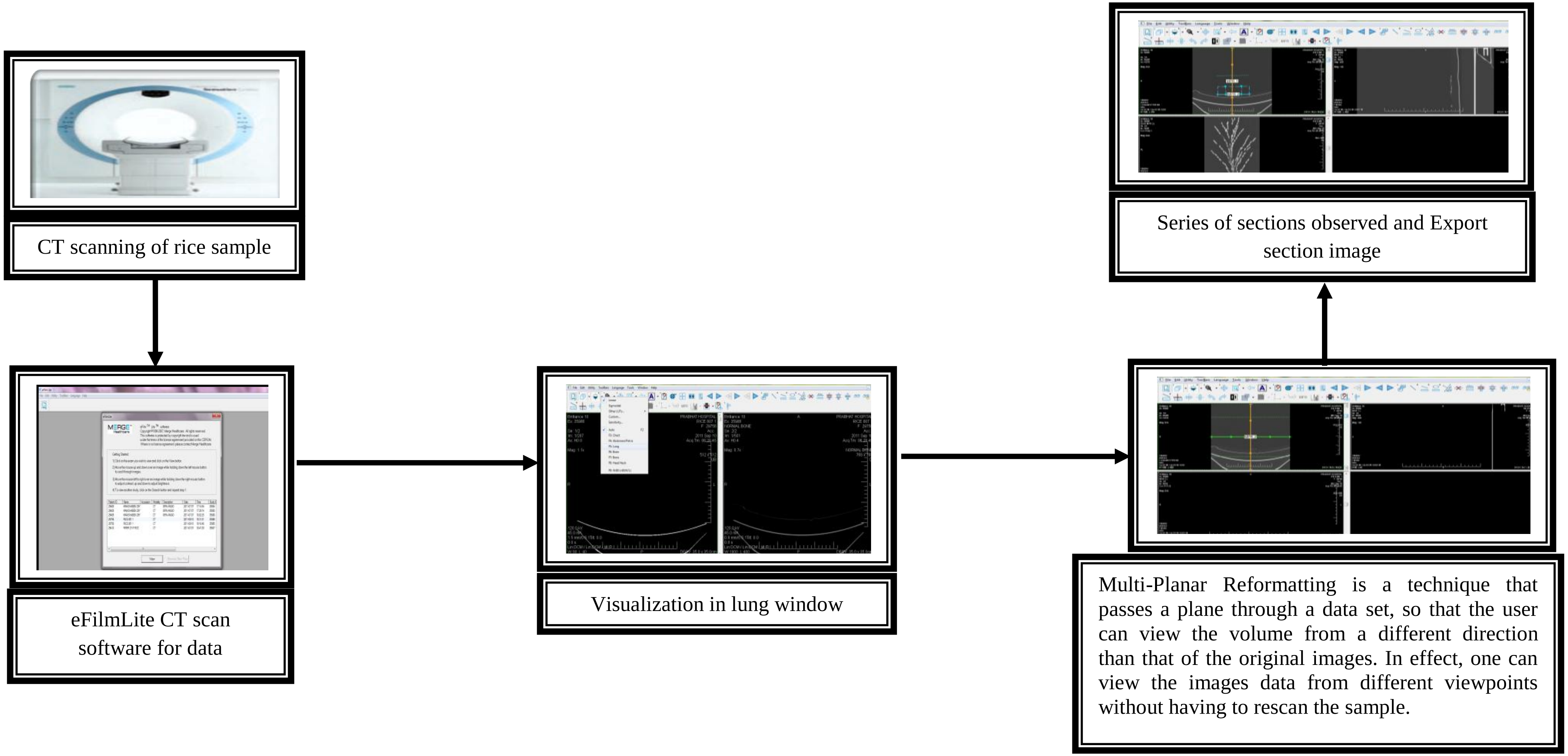

A schematic diagram of CT scan procedure for rice panicle

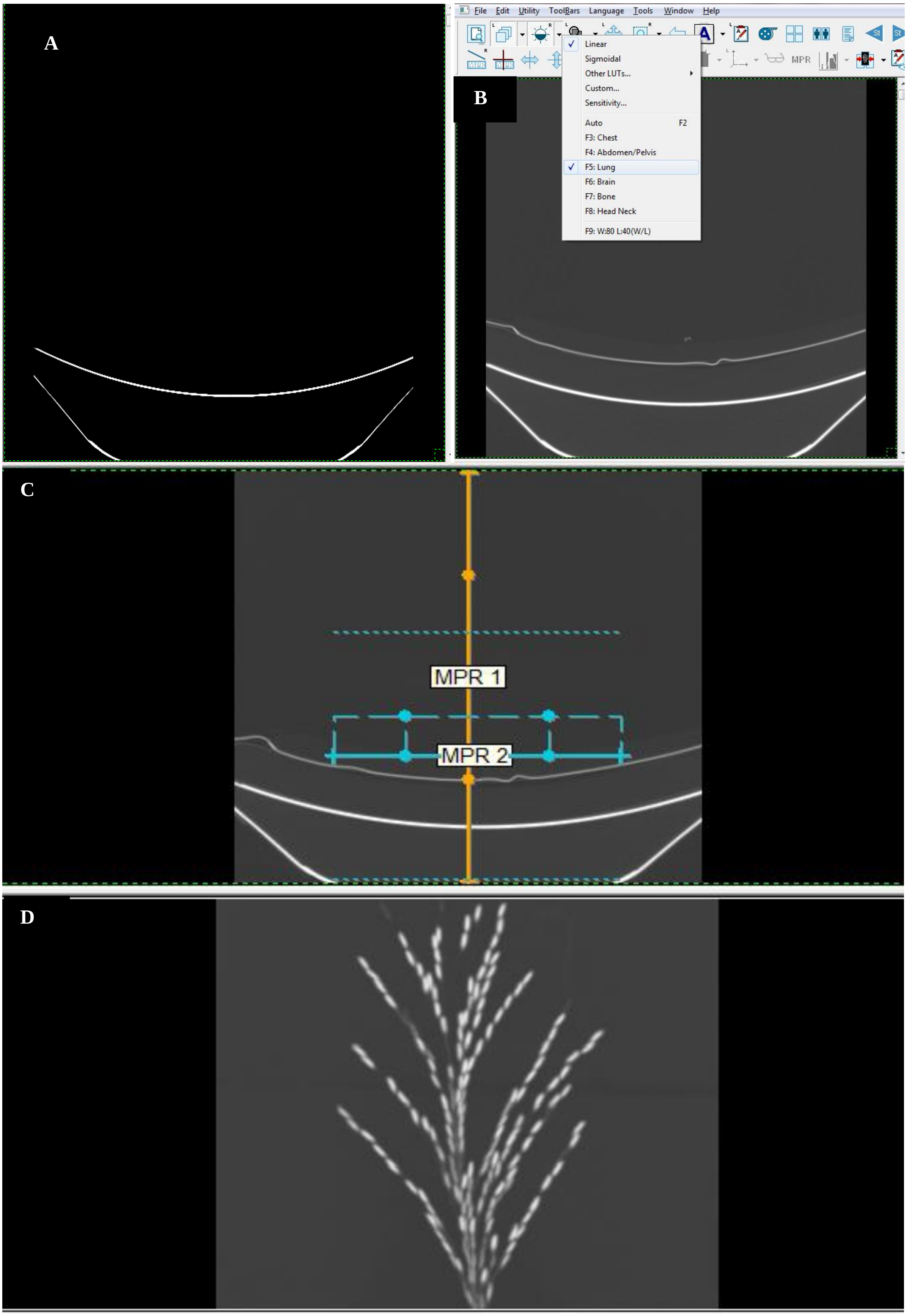

Supplementary Fig: 1 (A) eFilmLite CT scan software, (B) W/L (window / level) adjustment, (C) MPR (Multi-Planar Reformatting) tool and (D) Series of sections observed and Export selected image

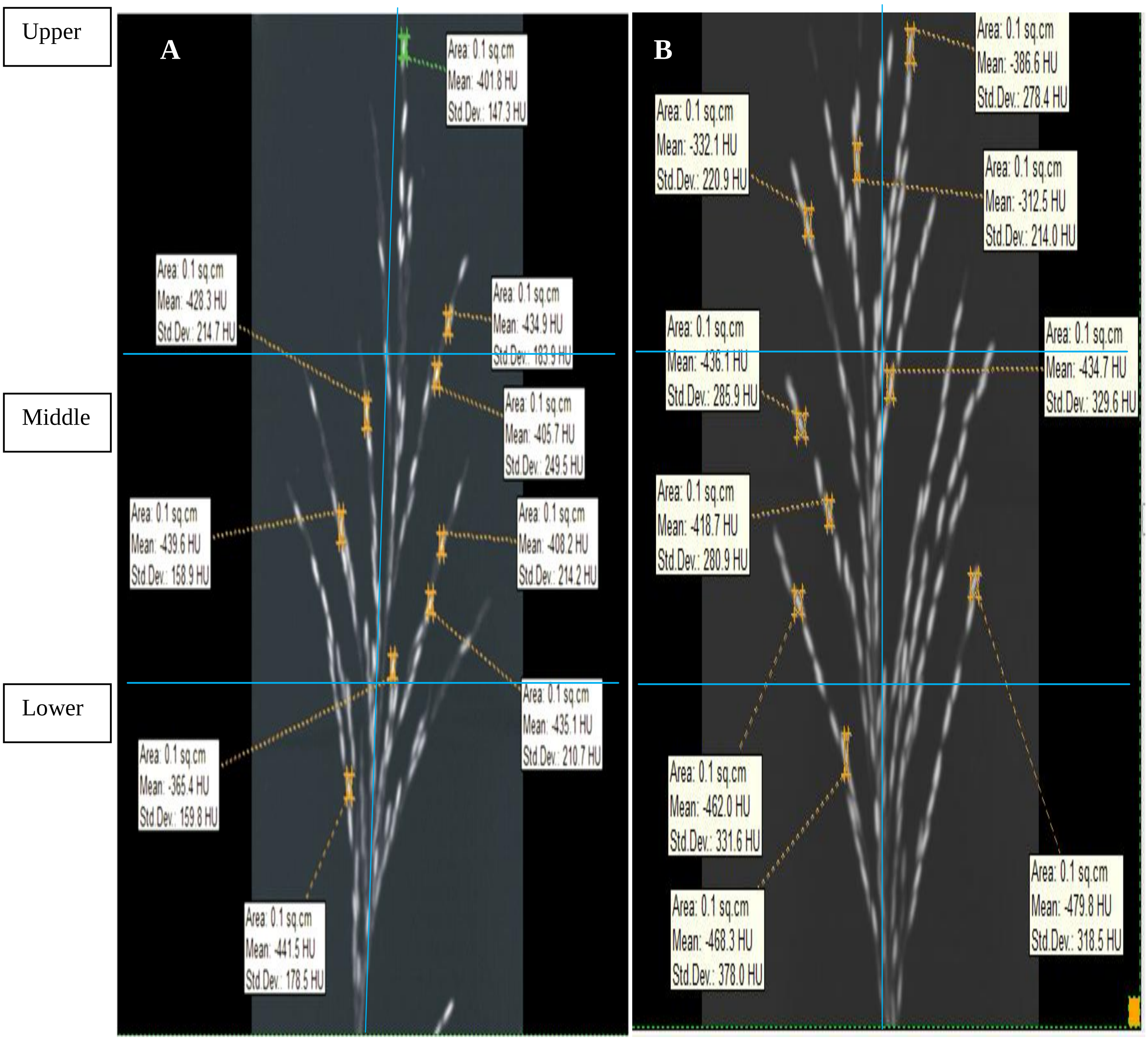

Supplementary Fig. 2. Calculation of HU value from the MPR images, (A) PAC801 and (B) PAC807. Rice panicle axis divided equally in to three part: upper, middle and lower in both the varieties (individual white portion in Figure indicates seeds)

.

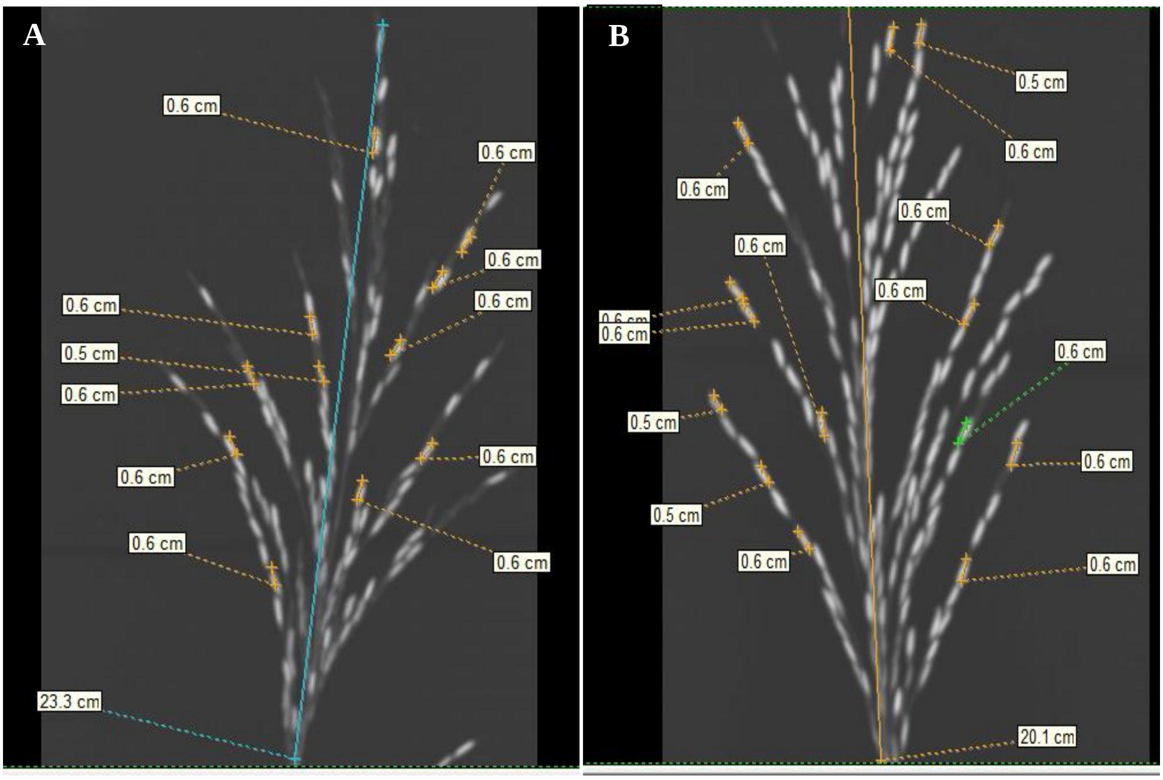

Supplementary Fig: 3 Seed size measurement in eFilmLite CT software (A) PAC801and (B) PAC 807

Supplementary Table: 1 seven rice varieties used in study

No	Varieties Name
1	Shahydari-3
2	Panvel-1
3	GR-11
4	Karjat-2
5	Panvel-7
6	GR-4
7	GR-3
